# Neuroblastoma-related severe hypoperfusion in the cerebellum of an infant: A case of opsoclonus-myoclonus syndrome

**DOI:** 10.22038/AOJNMB.2022.65833.1459

**Published:** 2023

**Authors:** Junki Takenaka, Kenji Hirata, Shiro Watanabe, Hideaki Shiraishi, Kohsuke Kudo

**Affiliations:** 1 Department of Diagnostic Imaging, Graduate School of Medicine, Hokkaido University, Sapporo, Japan; 2 Department of Nuclear Medicine, Hokkaido University Hospital, Sapporo, Japan; 3 Global Center for Biomedical Science and Engineering, Faculty of Medicine, Hokkaido University, Sapporo, Japan; 4 Department of Pediatrics, Graduate School of Medicine, Hokkaido University, Sapporo, Japan; 5 Department of Diagnostic and Interventional Radiology, Hokkaido University Hospital, Sapporo, Japan

**Keywords:** Opsoclonus- myoclonus syndrome, Neuroblastoma, Brain perfusion SPECT

## Abstract

A 2-year-old girl started to wobble without any specific triggers, so the patient was admitted to our hospital's pediatric department. The entire cerebellum showed severe atrophy on MRI and much lower uptake than that in the cerebral cortex on perfusion SPECT. The diagnosis of opsoclonus-myoclonus syndrome (OMS) was suspected. MRI visualized a small mass behind the inferior vena cava. Although its uptake on I-123 MIBG scintigraphy was inconclusive, the mass was surgically removed, and the diagnosis of neuroblastoma was pathologically confirmed. OMS is one of the paraneoplastic neurological syndromes with cerebellar ataxia, myoclonus of the trunk and extremities, and opsoclonus as its main symptoms. Approximately 50% of children cases with OMS are associated with neuroblastoma. The prognosis for neuroblastoma itself with OMS is relatively good, but the neurological prognosis is very poor. If there is decreased blood flow in the cerebellum of an infant, it may be necessary to search for neuroblastoma.

## Introduction

 Neuroblastoma is a common childhood tumor that arises from sympathetic nerve cells. OMS is a rare disorder of the nervous system with onset usually in the second year of life (1). This condition classically presents with a combination of characteristic eye movement disorder and myoclonus, in addition to ataxia, irritability, and sleep disturbance. It has been estimated that there is an underlying neuroblastoma in around 50% of children presenting with OMS (2). There are few reports of imaging findings in OMS. In this case, the entire cerebellum showed severe atrophy on MRI and almost no accumulation on perfusion SPECT.

## Case Report

**Figure 1:**
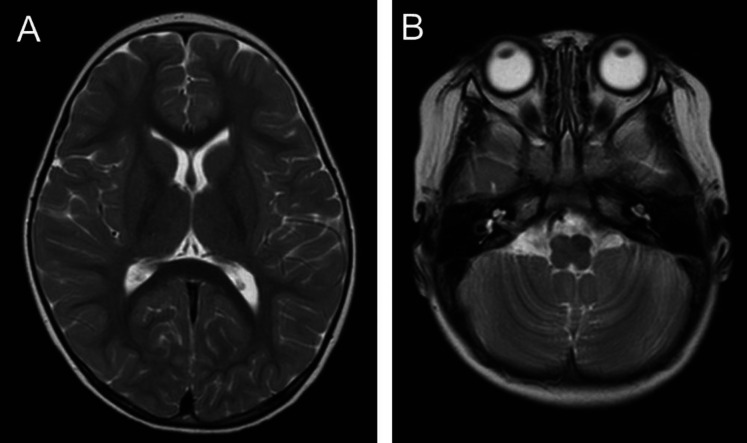
Brain MRI slices (**A**, cerebrum; **B**, cerebellum) acquired in the initial assessment did not show any abnormal findings

 A 2-year-old girl with no remarkable medical or family history was admitted to the pediatric department of a local hospital because she started to wobble without any specific triggers. She was unable to maintain standing and sitting positions and had tremor. No abnormality was noted on MRI of the brain (Figure 1) or laboratory tests. Because her symptoms did not improve after 2 months, the patient was transferred to our hospital's pediatric department.

**Figure 2:**
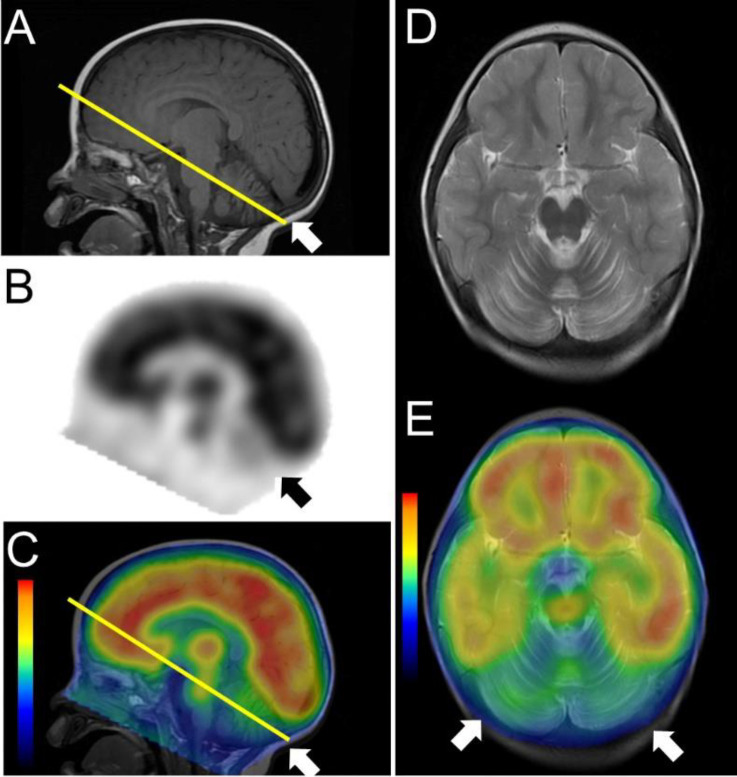
The entire cerebellum showed severe atrophy on Brain MRI (**A**), and much lower uptake than that in the cerebral cortex on perfusion SPECT and corresponding fused SPECT/MRI (**B**-**E**)

 A striking finding was observed on brain MRI and perfusion SPECT using Tc-99m ethyl cysteinate dimer (ECD). The entire cerebellum showed severe atrophy on MRI and much lower uptake than that in the cerebral cortex on SPECT (Figure 2). Myoclonus newly appeared during hospitalization. Considering cerebellar atrophy and hypoperfusion, myoclonus together, we suspected the diagnosis of opsoclonus-myoclonus syndrome (OMS).

**Figure 3:**
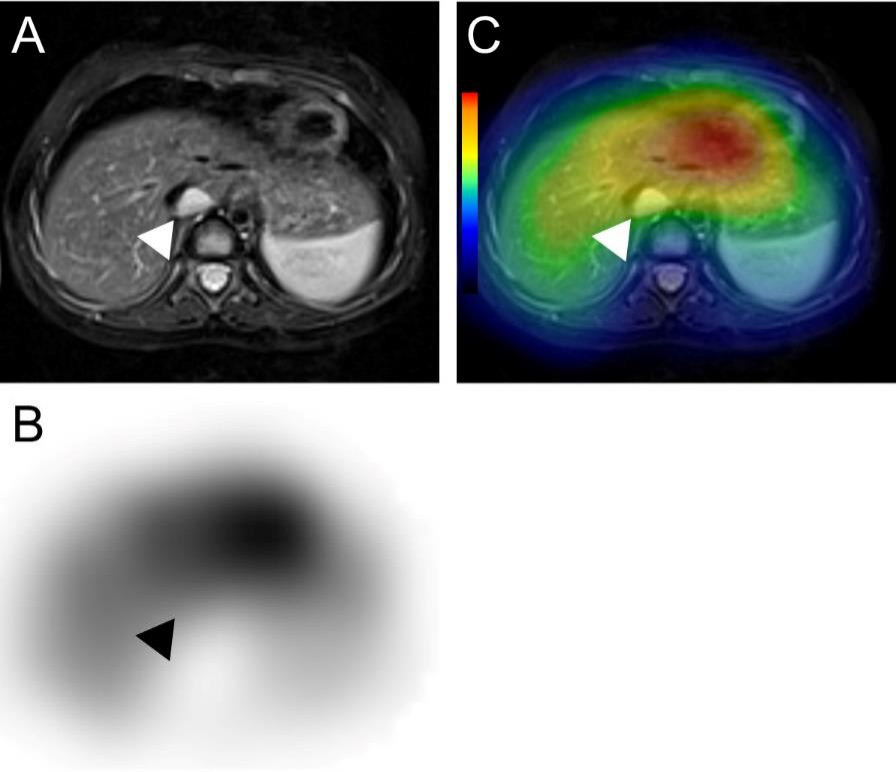
Fat suppressed T2-weighted MRI visualized a small mass behind the inferior vena cava (**A**). Its uptake was equal to the liver on I-123 MIBG scintigraphy (**B**-**C**)

 As OMS is often related to neuroblastoma (2), we performed whole-body surveillance. Fat suppressed T2-weighted MRI visualized a small mass behind the inferior vena cava (Figure 3, A). We further performed I-123 metaiodobenzyl-guanidine (MIBG) scintigraphy and found that the MIBG uptake in the tumor was comparable to that in the liver, although the small size of the lesion made it difficult to evaluate the true uptake in the tumor (Figure 3, B-C). The mass was surgically removed, and the diagnosis of neuroblastoma was pathologically confirmed. Because the clinical stage was considered to be International Neuroblastoma Staging System (INSS) stage 1, post-operative additional chemotherapy was omitted. She started to receive intravenous immunoglobulin (IVIg) before the operation and received steroid pulse therapy twice after the operation. Despite the treatments, her neurological symptoms including myoclonus persisted without exacerbation.

## Discussion

 OMS is one of the paraneoplastic neurological syndromes and is an extremely rare disease with cerebellar ataxia, myoclonus of the trunk and extremities, and opsoclonus as its main symptoms (3). It is different from para-neoplastic encephalitis. Children with OMS frequently have antibodies for central nervous system, and thus autoimmune mechanisms are believed to underlie in OMS. However, to our knowledge, no specific pathogenic auto-antibodies have been identified yet (2). The incidence rate is reported to be 0.18 cases per million per year, with an average patient age of 1.5 years (4). The cases of approximately 50% of children with OMS are associated with neuroblastoma (1) and approximately 2%–3% of children with neuroblastoma develop tumor-associated OMS (5). Neuroblastoma, a malignant tumor originating from the sympathetic nervous system, is the most common extra-cranial solid tumor in children, and accounts for up to 15% of cancer deaths in childhood (6). In this case, the diagnosis was INSS stage 1 neuroblastoma, for which the prognosis is good, and thus additional treatment was not performed after surgery. The prognosis for neuroblastoma itself with OMS is relatively good, but the neurological prognosis is very poor (7). In long-term follow-up after childhood OMS, cognitive and behavioral deficits can severely affect everyday function. Although the optimal treatment for OMS has not been established, immunotherapy including adrenocorticotropic hormone (ACTH) or corticosteroids, plasmapheresis, IV immunoglobulin (IVIg), and rituximab or cyclophosphamide is widespreadly used (2). In this case, IVIg and corticosteroid were used; however, the myoclonus has remained.

 Although there are few reports of imaging findings in OMS, it was reported that MRI did not show abnormal findings in the acute phase but, in the chronic phase, cerebellar atrophy became apparent (8). Another case report using SPECT described an increase in cerebellar blood flow in the acute phase and a decrease in the chronic phase (9). In this case, as the entire cerebellum showed severe atrophy on MRI and much lower uptake than that in the cerebral cortex on perfusion SPECT, her syndrome was already considered in the chronic phase. If decreased cerebellar blood flow is observed in an infant with OMS, it may be necessary to search the entire body for neuroblastoma.
